# Calculation of delivered composite dose from Calypso tracking data for prostate cancer: And subsequent evaluation of reasonable treatment interruption tolerance limits

**DOI:** 10.1002/acm2.12684

**Published:** 2019-07-29

**Authors:** Hui Zhao, Vikren Sarkar, Brian Wang, Prema Rassiah‐Szegedi, Martin Szegedi, Y. Jessica Huang, Long Huang, Jonathan Tward, Bill Salter

**Affiliations:** ^1^ University of Utah Salt Lake City UT USA; ^2^ Yale University New Haven CT USA

**Keywords:** Calypso, Calypso tolerance level, composite dose, PTV margin

## Abstract

**Purpose:**

In this study we calculate composite dose delivered to the prostate by using the Calypso tracking ‐data‐ stream acquired during patient treatment in our clinic. We evaluate the composite distributions under multiple simulated Calypso tolerance level schemes and then recommend a tolerance level.

**Materials and methods:**

Seven Calypso‐localized prostate cancer patients treated in our clinic were selected for retrospective analysis. Two different IMRT treatment plans, with prostate PTV margins of 5 and 3 mm respectively, were computed for each patient. A delivered composite dose distribution was computed from Calypso tracking data for each plan. Additionally, we explored the dosimetric implications for “worst case” scenarios by assuming that the prostate position was located at one of the eight extreme corners of a 3 or 5 mm “box.” To characterize plan quality under each of the studied scenarios, we recorded the maximum, mean, and minimum doses and volumetric coverage for prostate, PTV, bladder, and rectum.

**Results and discussions:**

Calculated composite dose distributions were very similar to the original plan for all patients. The difference in maximum, mean, and minimum doses as well as volumetric coverage for the prostate, PTV, bladder, and rectum were all < 4.0% of prescription dose. Even for worst scenario cases, the results show acceptable isodose distribution, with the exception for the combination of a 3 mm PTV margin with a 5 mm position tolerance scheme.

**Conclusions:**

Calculated composite dose distributions show that the vast majority of dosimetric metrics agreed well with the planned dose (within 2%). With significant/detrimental deviations from the planned dose only occurring with the combination of a 3 mm PTV margin and 5 mm position tolerance, the 3 mm position tolerance strategy appears reasonable, confirming that further reducing prostate PTV margins to 3 mm is possible when using Calypso with a position tolerance of 3 mm.

## 1 INTRODUCTION

Intensity‐modulated radiation therapy (IMRT) treatment for prostate cancer has gained in popularity over the last two decades. Compared to three‐dimensional conformal radiation therapy (3DCRT), IMRT has been proven to maintain the same level of tumor control probability (TCP) while decreasing normal tissue toxicity. One important advantage of the IMRT technique is the ability to shape dose distributions, thus avoiding nearby critical structures such as bladder and rectum.^1^, ^2^, ^3^

Due to the high cumulative dose levels typically delivered to the prostate, and its proximity to sensitive normal structures, it is important to accurately locate the IMRT‐shaped dose distribution. Image‐guided radiation therapy (IGRT) has evolved as a valuable tool in achieving an accurate placement of such shaped, high dose level distributions. There are several prostate IGRT techniques widely used for prostate treatments, including implanted fiducial markers, ultrasound, cone‐beam CT, and Calypso four‐dimensional (4D) localization system. The prostate treatment margins could be reduced due to IGRT technique.^4^, ^5^, ^6^

Calypso is a prostate target positioning technique which can be used to directly localize and track prostate position during external beam radiation therapy with a precision of about 0.5 mm.^7^, ^8^, ^9^, ^10^ By tracking the prostate's position in real time during treatment, intrafraction motion‐induced errors can be reduced significantly by interrupting treatment and correcting prostate position alignment; thus ensuring accurate delivery of conformal prostate treatment. To use Calypso in this fashion tolerance limits must be defined, beyond which the treatment is interrupted and prostate alignment is corrected. Commonly employed clinical tolerance limits for Calypso are 3–5 mm, based largely on vendor recommendations. Literature providing dosimetric rationale for a given tolerance scheme strategy is currently lacking. Given the real‐time tracking data stream describing prostate position provided by Calypso, it is theoretically possible to retrospectively calculate an accurate representation of actual dose delivered each day, including the effects of during‐treatment motion‐induced errors. Various tolerance levels can be assumed/simulated during such a calculation and, therefore, a dosimetric rationale can be evaluated/ developed to determine reasonable Calypso tolerance levels for intrafraction motion limitation in prostate.

Traditionally, prostate margins have been 1 cm for 3DCRT, and were subsequently reduced to 5 mm with the introduction of IGRT. The possibility of further reducing margins is the subject of several current investigations.^11^, ^12^, ^13^

In this study we calculate composite dose delivered to the prostate by using the Calypso tracking‐data‐stream acquired during each of the 42 fractions of seven prostate patients treated in our clinic (294 total fractions). We then evaluate the composite distributions that would have been delivered under multiple simulated Calypso tolerance level schemes and to recommend a tolerance level strategy that is based on achievable delivered dose distribution.

## 2 MATERIALS AND METHODS

**Figure 1:**
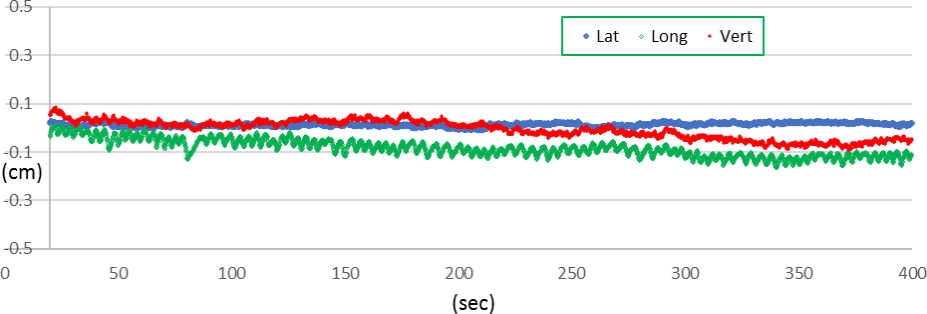
A typical Calypsotracking data during patient radiation treatment.

For the purposes of this study, a total of seven Calypso‐localized prostate cancer patients treated in our clinic were randomly selected for retrospective analysis with IRB approval. Each patient was prescribed 75.6 Gy in 42 fractions. Two different IMRT treatment plans, with prostate PTV margins of 5 and 3 mm respectively, were computed for each of seven patients. The PTV margin of 5 mm was chosen because it is the default tolerance value in the Calypso software and it represents a commonly employed prostate PTV margin when using Image‐Guided techniques^14^, ^15^ and the prostate PTV margin of 3 mm was chosen because it equals the treatment interruption tolerance value currently used in our clinic. The tolerance of 3 mm was initially chosen in our clinic only because we observed for our early patients that this level was achievable without excessive treatment interruption. A composite dose distribution was computed for each plan in the treatment planning system (Corvus, version 8, Best NOMOS, Pittsburg, PA) to intentionally misplace the dose distribution for each of the 42 daily fractions by the amount that the patient's prostate was observed to be out of position for that particular day. The Calypso system's continuous temporo‐positional data stream was used to calculate the average x, y, and z locations of the prostate while the treatment beam was on for that day, with an inherent assumption that it remained at that position for that day's entire treatment. This approach was reasonable because we observed similar intrafractional motion pattern of the prostate during beam on time, and the averaged position could reasonably represent the prostate position during the entire treatment. (Fig. 1 showed a typical Calypso tracking data during radiation treatment.) In this manner we could calculate a reasonable estimation of the total delivered dose for this patient, for each of the two PTV margin scenarios.

Additionally, we endeavored to explore the dosimetric implications of utilizing either the 3 mm tolerance scheme or the 5 mm tolerance scheme. For each of these tolerance schemes we explored the worst case scenario by assuming that on each given day of the 42 fraction Calypso‐guided treatment course the prostate position was located at one of the eight extreme corners of the 3 or 5 mm “box” defined by the respective tolerance scheme (e.g., *x* = +3, *y* = +3, *z* = +3; *x* = −3, *y* = +3, *z* = +3; *x* = −3, *y* = −3, *z* = +3; and so on for all eight “corners”). By uniformly distributing the prostate positional error between the eight possible worst case locations, we developed a composite plan that was representative of a reasonable “worst case” delivered dose distribution for each tolerance scenario, for both the 3 and 5 mm PTV margin plans, for each patient.

To characterize plan quality under each of the studied scenarios, we recorded the maximum, mean, and minimum doses for prostate, PTV, bladder, and rectum. The volumetric coverage of prostate, PTV, bladder, and rectum for 100% and 95% prescription dose (71.82 Gy) were calculated, along with the volume coverage of bladder and rectum for 65 and 40 Gy two dose levels. The reason for choosing 65 and 40 Gy dose levels for volume coverage of bladder and rectum is that these two dose levels are reasonably used for normal tissue tolerance analysis of prostate treatment plan.

## 3 RESULTS AND DISCUSSIONS

### 3.A Composite dose calculation

**Figure 2:**
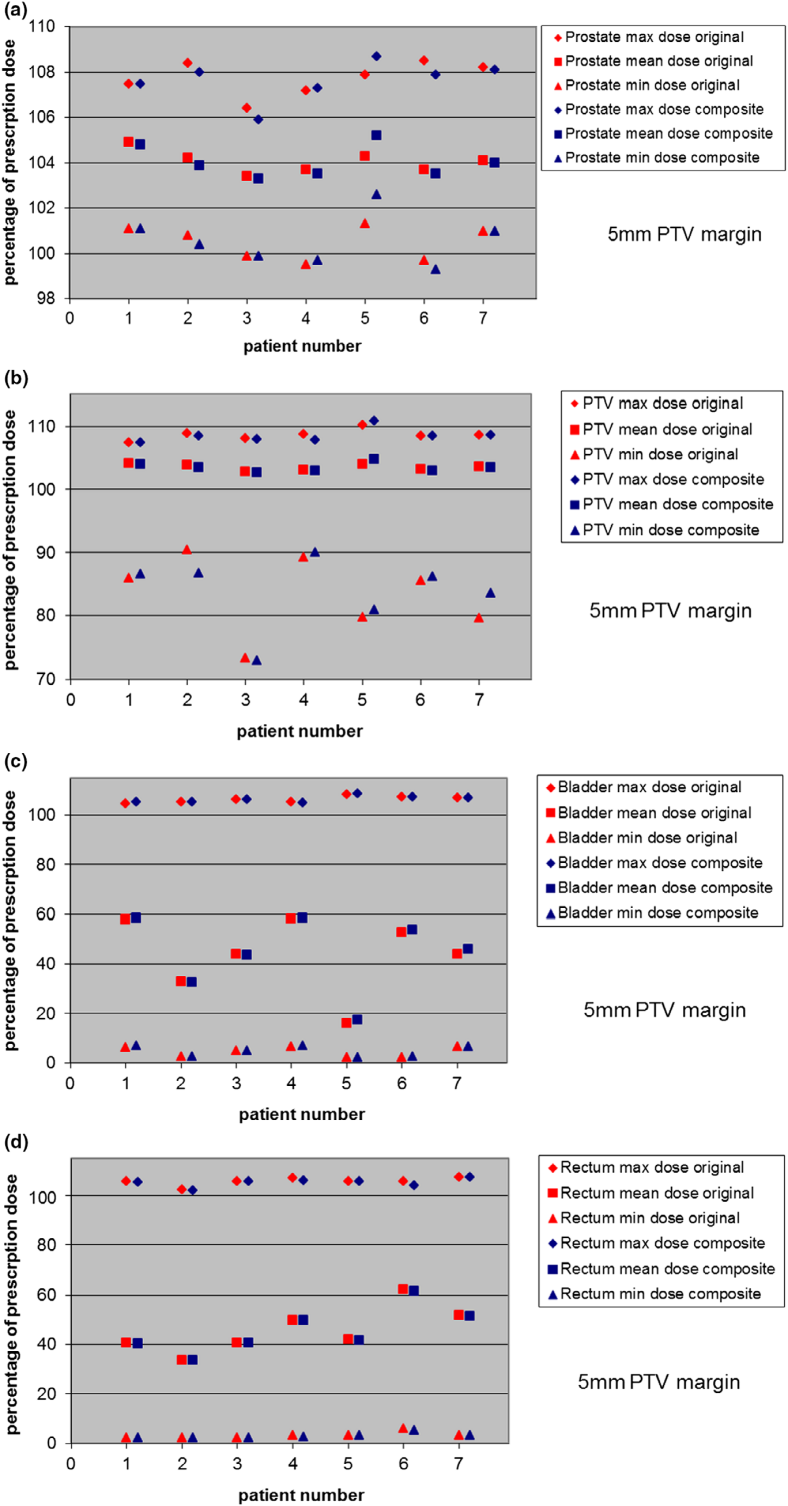
Composite delivered dose using Calypsotracking data. Target and critical structure doses are compared with the original plan for 5 mm PTV margins for all seven patients. The composite plan is the sum of 42 fractions of actual treatment, average prostate position for each day. The maximum, mean, and minimum dose values are presented as the percentage of prescription dose. The top figure shows dose comparison for prostate, the second figure shows dose comparison for PTV, the third figure shows dose comparison for bladder, and the bottom figure shows dose comparison for rectum.

**Figure 3:**
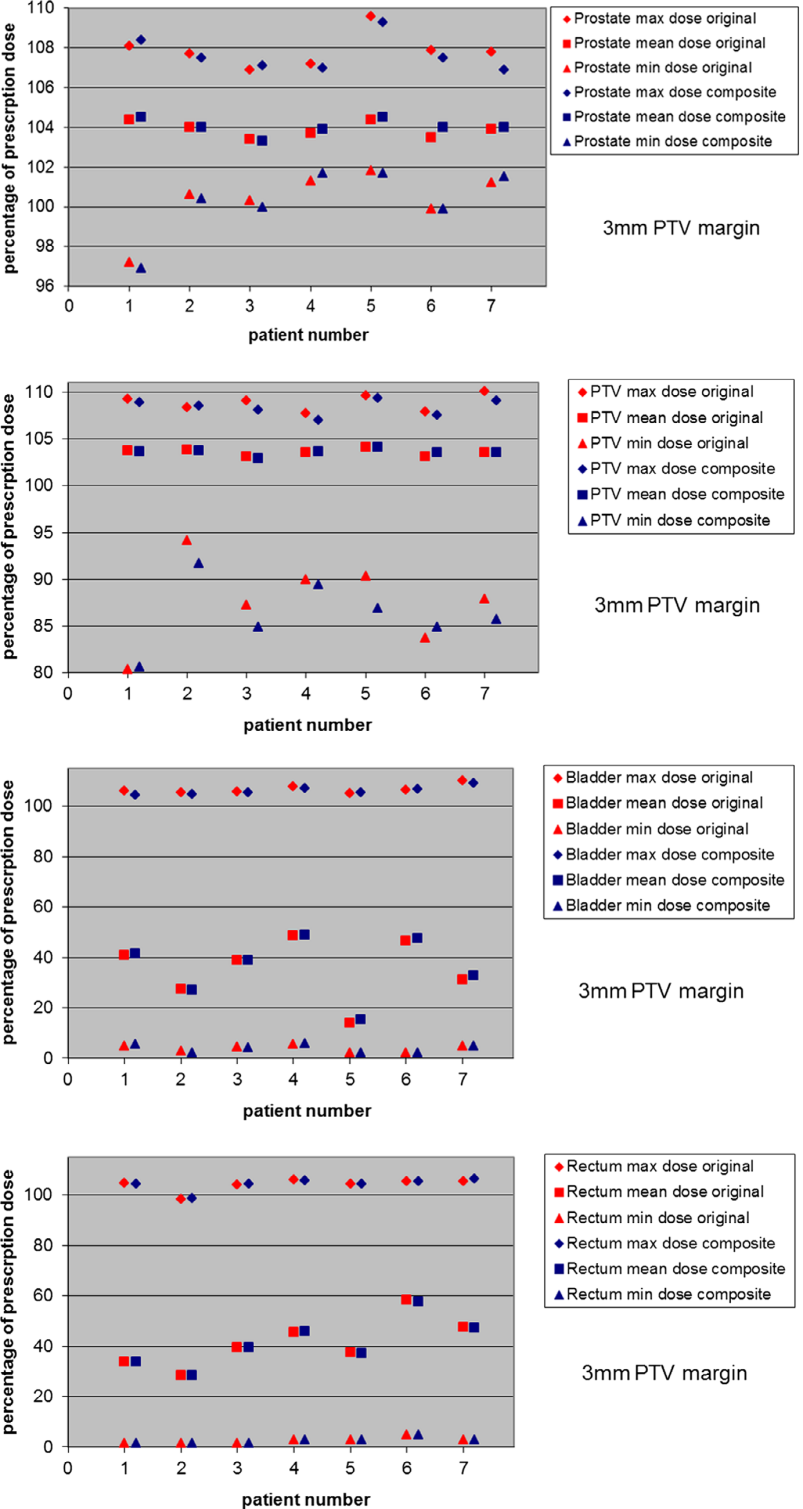
Composite delivered dose using Calypsotracking data. Target and critical structure doses are compared with the original plan for 3 mm PTV margins for all seven patients. The composite plan is the sum of 42 fractions of actual treatment, average prostate position for each day. The maximum, mean, and minimum dose values are presented as the percentage of prescription dose. The top figure shows dose comparison for prostate, the second figure shows dose comparison for PTV, the third figure shows dose comparison for bladder, and the bottom figure shows dose comparison for rectum.

The averaged location of the prostate (while the treatment beam was on of that day) was calculated from the Calypso system's continuous tempero‐positional data stream.The values of x, y, and z were in the range of 0 to 3 mm, since the Calypso tolerance level was set to 3 mm and any excursion above that limit led to the patient being repositioned. Composite dose calculations based on Calypso tracking data of 42 fractions for both 5 and 3 mm PTV margins were very similar to original plan for all patients. The difference in maximum, mean, and minimum doses for prostate, PTV, bladder, and rectum were all <2.0% of prescription dose (Figs. 2 and 3), with the exception of the difference of minimum dose to the PTV which was within 4.0%. The volumetric coverage difference for the prostate, PTV, bladder, and rectum for 100% and 95% prescription dose was less than 2.5%, as was the volumetric coverage of bladder and rectum at the 65 and 40 Gy levels. This essentially confirms that, when using a sophisticated, intrafractional tracking system to accurately maintain target position, an accurate delivered dose distribution can be achieved.

### 3.B 3 and 5 mm Tolerance Scheme — worst case dose calculation

**Figure 4:**
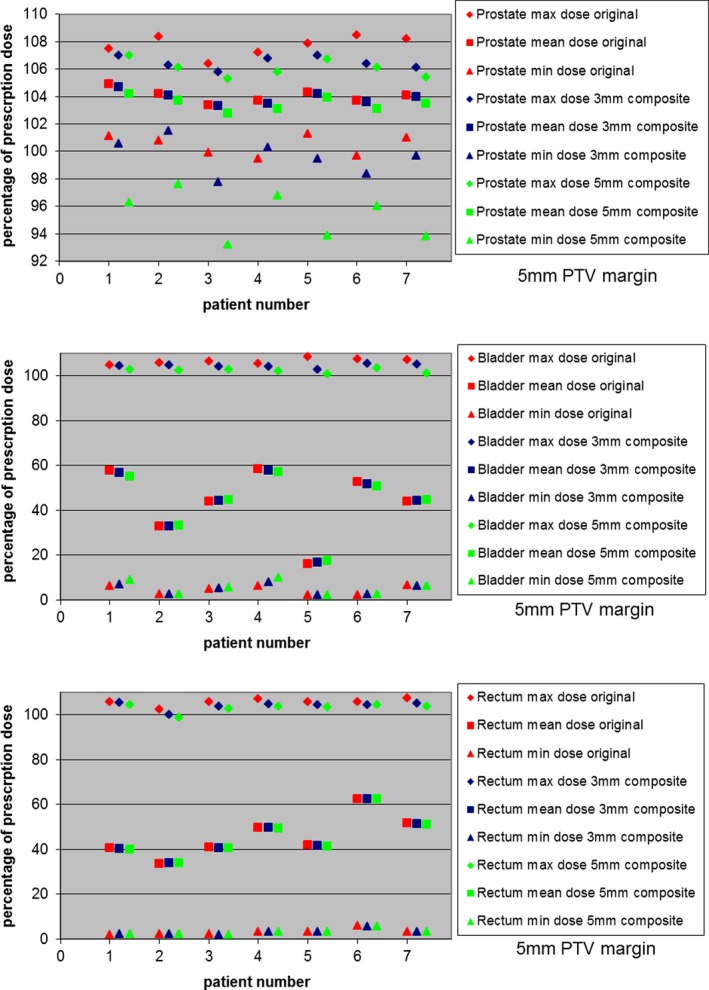
Prostate and critical structures dose comparison of original and composite plans for 5 mm PTV margin, for both 5 and 3 mm Tolerance Schemes for all seven patients. The composite plans are the sum of the eight worst case combinations of Calypso tolerance shifts of 3 and 5 mm, respectively. The maximum, mean, and minimum dose values are presented as the percentage of prescription dose. The top figure shows dose comparison for prostate, the middle figure shows dose comparison for bladder, and the bottom figure shows dose comparison for rectum.

**Figure 5:**
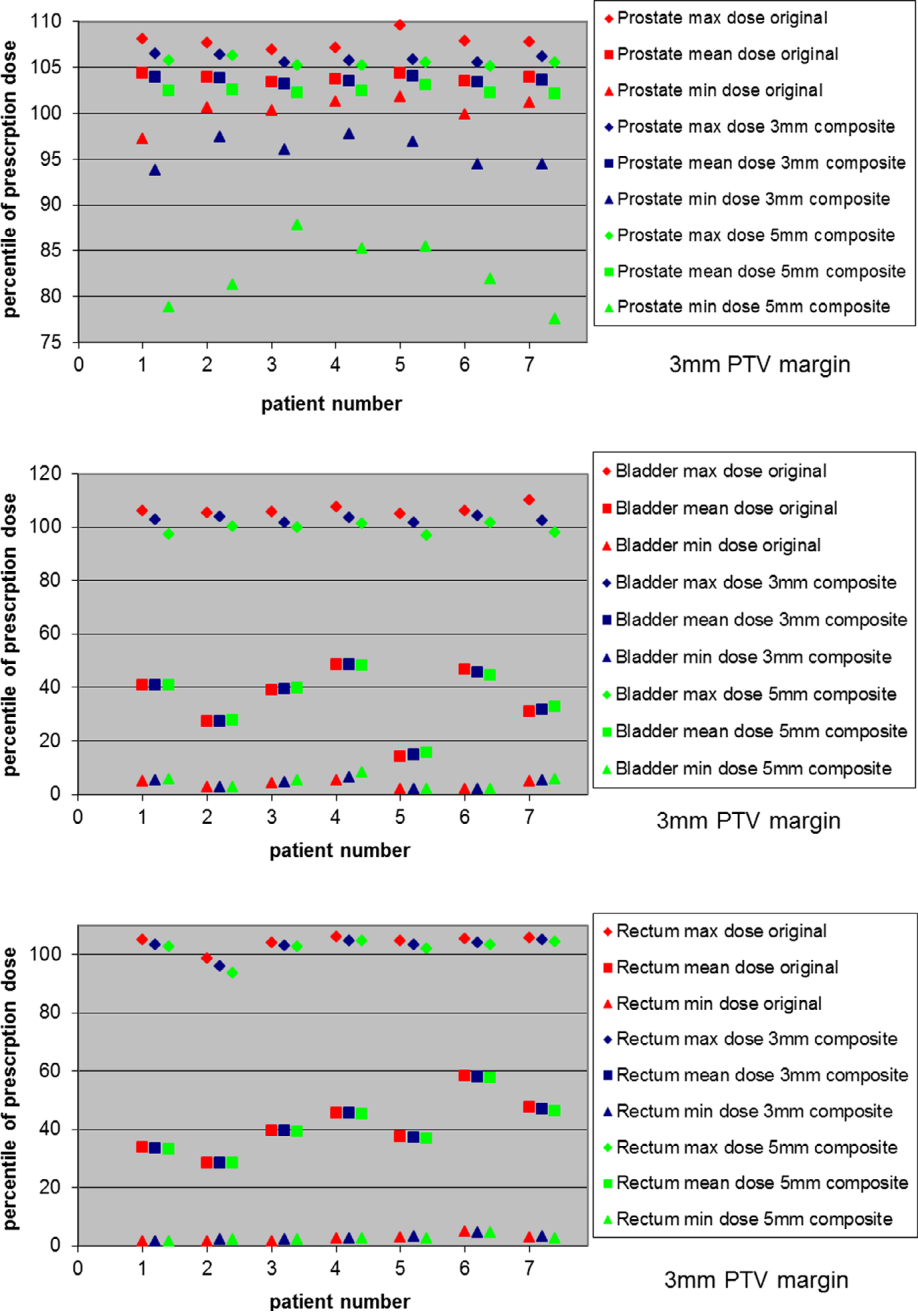
Prostate and critical structures dose comparison of original and composite plans for 3 mm PTV margin, for both 5 and 3 mm Tolerance Schemes for all seven patients. The composite plans are the sum of the eight worst case combinations of Calypso tolerance shifts of 3 and 5 mm, respectively. The maximum, mean, and minimum dose values are presented as the percentage of prescription dose. Note that prostate, Bladder and rectum dosing is similar to the original plan except for the combination of 3 mm PTV margin with 5 mm Tolerance Scheme. The top figure shows dose comparison for prostate, the middle figure shows dose comparison for bladder, and the bottom figure shows dose comparison for rectum

The difference between the original calculated treatment plan and the worst‐case delivered dose scenarios is shown in Figs. 4 and 5 and Tables 1 and 2 for each tolerance scheme for all patients, for both 5 and 3 mm PTV margin plans. Doses are reported as percentage of prescribed dose and as percentage volume covered by the specified dose levels, respectively.

**Table 1:** Target volumetric coverage comparison of original and composite plans for 5 and 3 mm Tolerance Scheme, for both 5 and 3 mm PTV margins for all seven patients. The composite plans are the sum of the eight worst case combinations of Calypso tolerance shifts of 3 and 5 mm, respectively. The values are reported as the percentage volume covered by the specified dose levels. The full treatment prescription was 75.6 Gy

Prostate	Patient number		1	2	3	4	5	6	7
5 mm PTV margin	75.6 Gy	Original	100	100	100	99.99	100	99.98	100
		3 mm tolerance composite	0	0	−0.15	+0.01	0	−0.05	0
		5 mm tolerance composite	−0.52	−0.45	−3.17	−0.62	−0.79	−1.26	−1.49
	71.82 Gy	Original	100	100	100	100	100	100	100
		3 mm tolerance composite	0	0	0	0	0	0	0
		5 mm tolerance composite	0	0	−0.10	0	0	0	−0.01
3 mm PTV margin	75.6 Gy	Original	99.79	100	100	100	100	100	100
		3 mm tolerance composite	−1.24	−0.03	−0.39	−0.20	−0.05	−0.52	−1.16
		5 mm tolerance composite	−11.22	−8.40	−10.25	−8.09	−6.47	−9.67	−11.66
	71.82 Gy	Original	100	100	100	100	100	100	100
		3 mm tolerance composite	−0.05	0	0	0	0	0	0
		5 mm tolerance composite	−2.84	−1.02	−1.26	−2.17	−0.42	−1.28	−5.19

Red and blue represents the % number was out of 10% and 5%.

**Table 2:** Critical structures volumetric coverage comparison of original and composite plans for 5 and 3 mm Tolerance Scheme, for both 5 and 3 mm PTV margins for all seven patients. The composite plans are the sum of the eight worst case combinations of Calypso tolerance shifts of 3 and 5 mm, respectively. The values are reported as the percentage volume covered by the specified dose levels. The full treatment prescription was 75.6 Gy. The top table shows dose comparison for bladder, and the bottom table shows dose comparison for rectum

Bladder	Patient number		1	2	3	4	5	6	7
5 mm PTV margin	65 Gy	Original	21.26	8.78	16.15	19.83	4.03	16.60	13.81
		3 mm tolerance composite	−4.51	−1.11	−3.34	−2.03	−1.18	−1.73	−2.40
		5 mm tolerance composite	−10.63	−2.70	−7.92	−5.14	−2.42	−4.67	−6.67
	40 Gy	Original	53.21	26.31	39.61	59.82	10.35	49.20	38.05
		3 mm tolerance composite	−1.05	−0.99	−0.60	−3.25	−0.55	−1.14	−1.70
		5 mm tolerance composite	−2.05	−2.42	−1.07	−6.84	−0.70	−2.41	−2.97
3 mm PTV margin	65 Gy	Original	8.43	6.20	11.61	14.20	2.31	10.78	8.82
		3 mm tolerance composite	−3.46	−0.94	−3.08	−1.93	−0.73	−1.38	−3.44
		5 mm tolerance composite	−6.89	−2.44	−7.10	−5.50	−1.53	−3.98	−7.13
	40 Gy	Original	29.82	20.94	33.46	43.64	7.60	39.05	25.29
		3 mm tolerance composite	−1.20	−1.06	−0.73	−0.93	−0.26	−1.13	−2.12
		5 mm tolerance composite	−2.20	−2.52	−1.66	−2.94	−0.43	−2.67	−2.95

Rectum	Patient number		1	2	3	4	5	6	7
5 mm PTV margin	65 Gy	Original	14.66	8.82	16.56	20.87	11.12	25.87	23.68
		3 mm tolerance composite	−0.96	−0.58	−0.32	−0.57	−0.90	−0.23	−0.39
		5 mm tolerance composite	−2.69	−1.34	−1.36	−3.48	−2.35	−1.46	−1.26
	40 Gy	Original	33.28	33.61	38.14	51.59	38.60	65.59	53.33
		3 mm tolerance composite	−0.49	−1.19	+0.11	−0.39	+0.32	−0.15	−0.94
		5 mm tolerance composite	−0.61	−2.91	−0.08	−1.56	−0.06	−0.32	−2.49
3 mm PTV margin	65 Gy	Original	9.44	3.86	14.19	16.44	7.04	23.17	21.65
		3 mm tolerance composite	−0.90	0	−0.43	−0.98	−0.56	−0.61	−1.09
	40 Gy	5 mm tolerance composite	−2.51	−0.36	−1.72	−2.24	−1.71	−1.96	−2.76
		Original	25.30	27.27	37.38	47.53	31.88	61.08	48.49
		3 mm tolerance composite	−0.38	−0.82	+0.57	−0.53	−0.45	+0.12	−1.53
		5 mm tolerance composite	−0.99	−2.29	+0.69	−2.07	−1.30	−1.20	−4.08

Red and blue represents the % number was out of 10% and 5%.

As seen in Figs. 4 and 5, for both 5 and 3 mm PTV margin, for both 3 and 5 mm tolerance levels, the maximum and mean prostate dose difference from original plan was within 2%, and the maximum and mean dose difference for both bladder and rectum was ranged from +1.7% to −12%, thus indicating acceptable isodose distribution for both tolerance scenarios. The only exception was for the, perhaps ill‐advised, combination of a 3 mm PTV margin with a 5 mm Tolerance Scheme, where minimum prostate dose could be as low as 77.6%. Of course, this assumes that the target is always at an extreme location within the tolerance “box.”

Table 1 shows the worst case combination volumetric coverage difference (from original plan) of prostate for 100% (75.6 Gy) and 95% prescription dose (71.82 Gy) for 5 and 3 mm PTV margin with combination of both 5 and 3 mm Tolerance Scheme. We can see from Table 1 that, with 5 mm PTV margin for both 5 and 3 mm Tolerance Scheme, the difference of volumetric coverage of prostate for 100% and 95% prescription dose was within 3.17%. With 3 mm PTV margin for 3 mm Tolerance Scheme, the difference of volumetric coverage of prostate was within 1.24%. We can also see from Table 1 that, even for the worst case scenario studied here, the only case in which prostate coverage is greatly compromised is for the combination of 3 mm PTV margin with 5 mm Tolerance Scheme (6.47% to 11.66% difference from the original plan).

Table 2 shows bladder and rectum volumetric coverage comparison of original and worst case combination composite plans for 5 and 3 mm Tolerance Scheme, for both 5 and 3 mm PTV margins, at the dose level of 65 and 40 Gy. We can see from Table 2, that most OAR volumetric doses at the two selected levels are reduced, relative to the original plan, for all PTV/ Worst‐Case‐Tolerance‐Scheme combinations simulated here. This seems reasonable when we recall that the “Worst Case” scenarios simulated here assumed the prostate to move equally between the extreme corners of the respective Tolerance geometric “box” being explored, thus serving to “wash out” or “blur” the dose distribution of extreme OAR dose values.

The decision to only investigate the vendor recommended 3 and 5 mm tolerances instead of smaller values was made for two reasons. Firstly, even for the worst case scenario where the prostate was at the extreme of 3 mm in all directions, the dosimetric evaluation still showed that the ensuing dose distribution was within 2% of the planned distribution. While a smaller tolerance for positioning within Calypso would ensure a higher fidelity of the delivered dose distribution to the planned distribution, this would also cause for many more treatment interruptions due to the prostate moving out of these smaller margins. That would likely cause extended treatment times which would, in turn, lead to a higher likelihood of motion.^16^, ^17^ With the 3 mm tolerances showing an adequate compromise between dose fidelity and treatment length, this was the lower limit used.

## 4 CONCLUSION

A clinically realistic calculation of delivered composite dose can be calculated using the averaged x, y, and z target position temporal data stream provided by Calypso. Calculated composite dose distributions show that, for the patients studied here, the vast majority of dosimetric metrics agreed quite well with predicted dose (i.e., within 2%), thus confirming the dosimetric value of during treatment target tracking.

A study of the dosimetric ramifications of two different treatment interruption Tolerance Schemes (3 and 5 mm), under simulated “worst case” situations, showed that the only combination that resulted in significant/detrimental deviation from planned dose was for the, perhaps ill‐advised, combination of 3 mm PTV margin with 5 mm Tolerance Scheme. Otherwise, the 3 mm Tolerance Scheme strategy that we are currently employing appears reasonable, as does a 5 mm Tolerance Scheme scenario.

It shows that further reducing prostate margin to 3 mm is possible for Calypso tolerance level set up at 3 mm.

## CONFLICT OF INTEREST

The authors declare no conflict of interest.
